# Derivation and validation of an artificial intelligence-based plaque burden safety cut-off for long-term acute coronary syndrome from coronary computed tomography angiography

**DOI:** 10.1093/ehjci/jeaf121

**Published:** 2025-04-17

**Authors:** Sarah Bär, Juhani Knuuti, Antti Saraste, Riku Klén, Tanja Kero, Takeru Nabeta, Jeroen J Bax, Ibrahim Danad, Nick S Nurmohamed, Ruurt A Jukema, Paul Knaapen, Teemu Maaniitty

**Affiliations:** Turku PET Centre, Turku University Hospital and University of Turku, P.O.Box 52, Turku FI-20521, Finland; Department of Cardiology, Bern University Hospital Inselspital, Freiburgstrasse 20, 3010 Bern, Switzerland; Turku PET Centre, Turku University Hospital and University of Turku, P.O.Box 52, Turku FI-20521, Finland; Department of Clinical Physiology, Nuclear Medicine, and PET, Turku University Hospital, Turku, Finland; Turku PET Centre, Turku University Hospital and University of Turku, P.O.Box 52, Turku FI-20521, Finland; Heart Center, Turku University Hospital and University of Turku, Turku, Finland; Turku PET Centre, Turku University Hospital and University of Turku, P.O.Box 52, Turku FI-20521, Finland; Nuclear Medicine & PET, Department of Surgical Sciences, Uppsala University, Uppsala, Sweden; Department of Cardiology, Leiden University Medical Center, Leiden, The Netherlands; Department of Cardiology, Leiden University Medical Center, Leiden, The Netherlands; Department of Cardiology, Amsterdam UMC, Vrije Universiteit Amsterdam, Amsterdam, The Netherlands; Department of Cardiology, Amsterdam UMC, Vrije Universiteit Amsterdam, Amsterdam, The Netherlands; Department of Cardiology, Amsterdam UMC, Vrije Universiteit Amsterdam, Amsterdam, The Netherlands; Department of Cardiology, Amsterdam UMC, Vrije Universiteit Amsterdam, Amsterdam, The Netherlands; Turku PET Centre, Turku University Hospital and University of Turku, P.O.Box 52, Turku FI-20521, Finland; Department of Clinical Physiology, Nuclear Medicine, and PET, Turku University Hospital, Turku, Finland

**Keywords:** coronary computed tomography angiography, artificial intelligence, plaque burden, acute coronary syndrome

## Abstract

**Aims:**

Artificial intelligence (AI) has enabled accurate and fast plaque quantification from coronary computed tomography angiography (CCTA). However, AI detects any coronary plaque in up to 97% of patients. To avoid overdiagnosis, a plaque burden safety cut-off for future coronary events is needed.

**Methods and results:**

Percent atheroma volume (PAV) was quantified with AI-guided quantitative computed tomography in a blinded fashion. Safety cut-off derivation was performed in the Turku CCTA registry (Finland), and pre-defined as ≥90% sensitivity for acute coronary syndrome (ACS). External validation was performed in the Amsterdam CCTA registry (the Netherlands). In the derivation cohort, 100/2271 (4.4%) patients experienced ACS (median follow-up 6.9 years). A threshold of PAV ≥ 2.6% was derived with 90.0% sensitivity and negative predictive value (NPV) of 99.0%. In the validation cohort 27/568 (4.8%) experienced ACS (median follow-up 6.7 years) with PAV ≥ 2.6% showing 92.6% sensitivity and 99.0% NPV for ACS. In the derivation cohort, 45.2% of patients had PAV < 2.6 vs. 4.3% with PAV 0% (no plaque) (*P* < 0.001) (validation cohort: 34.3% PAV < 2.6 vs. 2.6% PAV 0%; *P* < 0.001). Patients with PAV ≥ 2.6% had higher adjusted ACS rates in the derivation [Hazard ratio (HR) 4.65, 95% confidence interval (CI) 2.33–9.28, *P* < 0.001] and validation cohort (HR 7.31, 95% CI 1.62–33.08, *P* = 0.010), respectively.

**Conclusion:**

This study suggests that PAV up to 2.6% quantified by AI is associated with low-ACS risk in two independent patient cohorts. This cut-off may be helpful for clinical application of AI-guided CCTA analysis, which detects any plaque in up to 96–97% of patients.

## Graphical Abstract

**Graphical Abstract:**
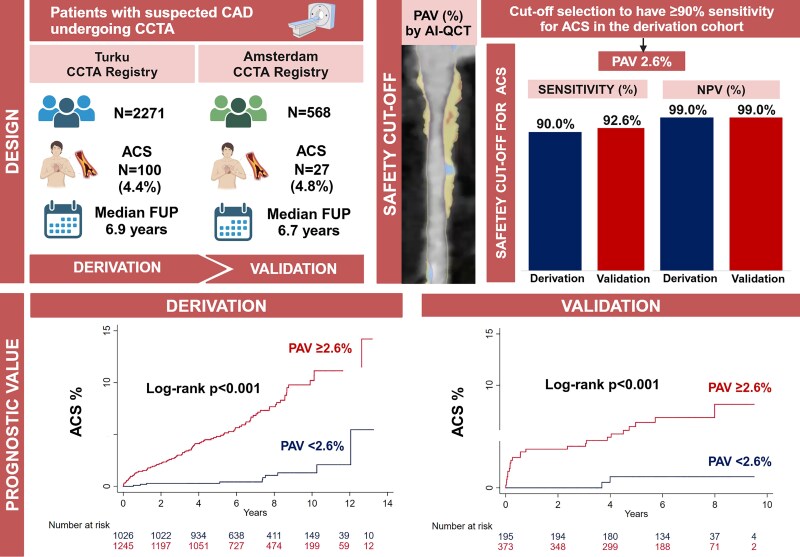

**See the editorial comment for this article ‘When coronary plaque is found in everyone: is anyone really at risk?’, by M. Guglielmo *et al*., https://doi.org/10.1093/ehjci/jeaf137.**

## Introduction

Coronary computed tomography angiography (CCTA) has emerged as one of the first-line non-invasive imaging methods for patients with the suspicion of coronary artery disease (CAD).^1,2^ Recent advances in CCTA using artificial intelligence (AI) have enabled accurate, automated, and fast plaque quantification and characterization.^3–6^ Therefore, the derivation of robust quantitative plaque cut-offs, potentially useful to inform clinical decision making, may have become feasible. Plaque burden quantified by AI has also proved prognostic value with continuously increasing event risk with increasing plaque burden.^7,8^ However, up to 97% of patients with a current indication for CCTA may present with any coronary plaque detected by AI.^7^ In order to avoid overdiagnosis, unnecessary downstream diagnostic testing, and implementation of advanced therapies in the broad range of patients undergoing CCTA, a clinically reasonable plaque burden safety cut-off for future coronary events is needed. Therefore, we aimed to derive and externally validate a plaque burden safety cut-off pre-defined to have ≥90% sensitivity for future acute coronary syndrome (ACS) among patients referred for CCTA using a previously validated AI-based plaque quantification and characterization tool with clearance from the US Food and Drug Administration.^3,4,9^

## Methods

### Derivation cohort

**Figure 1:**
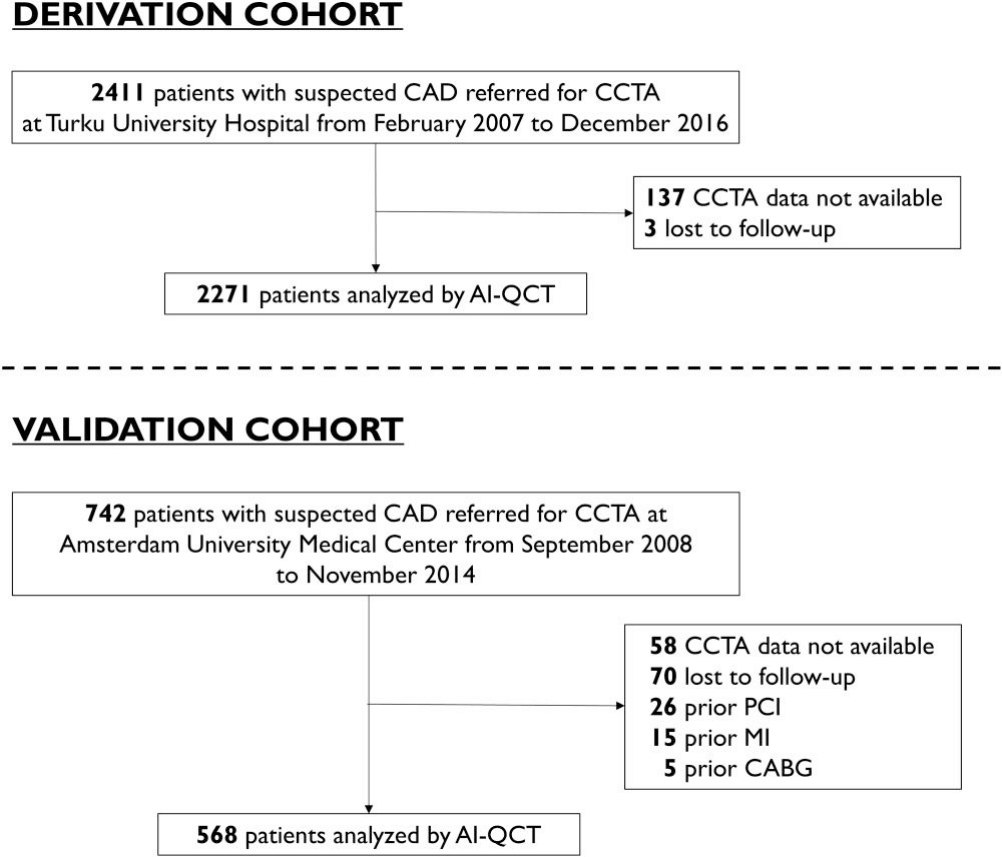
Patient flowchart. AI-QCT , artificial intelligence-guided quantitative computed tomography; CABG, coronary artery bypass grafting; CAD, coronary artery disease; CCTA, coronary computed tomography, angiography; MI, myocardial infarction; PCI, percutaneous coronary intervention.

For derivation, a consecutive cohort of 2411 symptomatic patients with suspected CAD having undergone CCTA at Turku University Hospital, Finland, from February 2007 to December 2016, was used. Out of these, 137 had non-retrievable CCTA images, and 3 patients were lost to follow-up, resulting in a final cohort of 2271 patients (*Figure 1*). Patients with previous coronary revascularization or known obstructive CAD were not considered for inclusion. Data on clinical characteristics, symptoms, and medication were retrospectively collected from electronic medical records.

### External validation cohort

Seven hundred and forty-two consecutive patients undergoing clinically indicated PET-CT at Amsterdam University Medical Center, the Netherlands, from September 2008 to November 2014 were used for validation. Of these, 58 had non-retrievable CCTA images, 70 were lost to follow-up, and 46 patients were excluded due to prior myocardial infarction (MI), percutaneous coronary intervention (PCI), or coronary artery bypass grafting, resulting in a total of 568 patients (*Figure 1*).

### Patient consent

The study complied with the Declaration of Helsinki. The Ethics Committees of the Hospital District of Southwest Finland and Amsterdam University Medical Center approved the study protocol and waived the need for informed consent.

### Clinical event adjudication

Comprehensive data on outcomes were collected using electronic medical records, standardized telephonic follow-up, and national registry databases. The identified events were confirmed by the investigators. ACS included MI or unstable angina pectoris (uAP) in accordance with the latest recommendations.^10^

### CCTA acquisition

CCTA imaging was performed as described previously.^11–13^ In brief, patients were targeted at a stable heart rate targeted below 65 bpm (either spontaneous or after administration of oral and/or intravenous metoprolol). Sublingual/oral nitrate was administered immediately before CCTA. Intravenously administered low-osmolal iodine contrast agents were used. Prospectively triggered CCTA acquisition was applied whenever feasible. At Turku University Hospital, a 64-row hybrid PET-CT scanner (GE Discovery VCT or GE D690, GE Healthcare, Waukesha, WI, USA) was used. The CCTA scans were performed with a collimation of 64 × 0.625 mm and gantry rotation time of 350 ms. The tube current was 600–750 mA and voltage 100–120 kV according to patient size. Iterative CCTA reconstruction with standard Kernel recommended by the vendor was applied. At Amsterdam University Medical Center, a 64-row hybrid PET-CT scanner (Philips Gemini TF 64, Philips Healthcare, Best, The Netherlands) or a 256-slice CT scanner (Philips Brilliance iCT, Philips Healthcare, Best, The Netherlands) was used. The CCTA scans were performed with the collimation of 64 × 0.625 mm, a gantry rotation time of 420 ms, a tube voltage of 120 kV, and a tube current of 800–1000 mA. Iterative CCTA reconstruction with standard Kernel recommended by the vendor was used.

### AI-guided quantitative computed tomography analysis

Artificial intelligence-guided quantitative computed tomography (AI-QCT) is a US Food and Drug Administration-cleared software service that uses a series of validated convolutional neural networks for image quality assessment, coronary segmentation and labelling, lumen wall evaluation, vessel contour determination, and plaque characterization. AI-QCT analysis was performed as described previously.^3,4^ In brief, coronary segments with a diameter ≥1.5 mm were included in the analysis using the 18-segment Society of Cardiovascular Computed Tomography model.^14^ Plaque volumes (cubic millimeter) were calculated for each coronary lesion and then summated to compute the total plaque volume at the patient level. Coronary plaque volume was normalized to the total per-patient vessel volume to account for variation in coronary artery volume, calculated as plaque volume (mm^3^)/vessel volume (mm^3^) × 100%, referred to as percent atheroma volume (PAV) (per cent). Plaque types were categorized using Hounsfield unit (HU) ranges, with non-calcified plaque volume (NCPV) defined as plaques with any component on a pixel-level basis with HU between −30 and +350, and calcified plaque volume (CPV) defined as >350 HU. Low-density non-calcified plaque volume (LD-NCPV) was defined as HU between −30 and +30.^15^ Owing to the low volume of LD-NCPV detected in these cohorts, consistent with previous observations,^7^ total NCPV was used for the prognostic models with continuous plaque burden. The prognostic value of LD-NCPV was assessed together with the presence of positive remodelling (i.e. lesion diameter/reference diameter ≥1.1), referred to as two feature positive plaque (two FPP).^15^ AI-QCT diameter stenosis was categorized by the Coronary Artery Disease-Reporting and Data System (CAD-RADS)^16^ and ≥50% stenosis classified as obstructive disease.

### Statistical analysis

Continuous variables are shown as mean ± standard deviation (SD) or median [IQR (25th–75th percentile)]. Categorical variables are shown as numbers with percentages. Mann–Whitney *U* test was used to compare continuous variables and two-sided χ^2^ test was used for categorical variables. The association between PAV, NCPV, and CPV per 1% increase and ACS was assessed with multivariable Cox regressions. Adjusting covariates were chosen based on clinical reasoning and consisted of age, sex, hypertension, diabetes mellitus, smoking, dyslipidaemia, family history of CAD, typical angina, and early elective coronary revascularization within 6 months from CCTA. Variables with a significant association in univariable models (i.e. age, sex, hypertension, typical angina, and early revascularization) were used as adjusting covariates (see Supplementary data online, *Table S1*). We constructed four multivariable reference models: clinical characteristics (Reference 1), CAD-RADS + Clinical characteristics (Reference 2), AI-QCT diameter stenosis ≥50% + Clinical characteristics, (Reference 3), and two FPP + Clinical characteristics (Reference 4). PAV, NCPV, and CPV per 1% were added to the reference models and the models’ performance was assessed and compared using Harrell’s C. For the model with FPP, only NCPV (%) without LD-NCPV (%) was added on top, since LD-NCPV (%) is part of the two FPP definitions. The binary plaque burden cut-off to predict ACS was chosen from receiver operating characteristic (ROC) curve analysis to have a sensitivity of ≥90% for ACS in the derivation cohort. A threshold selection according to the method of Youden, which gives equal weight to sensitivity and specificity^17^ is not suitable for derivation of a safety cut-off for adverse patient outcome, where sensitivity must be emphasized.^18^ However, for conceptual validation and comparison to previous work,^5^ we also tested a threshold according to Youden in total plaque volume. We then calculated sensitivity, negative predictive value (NPV), specificity, positive predictive value (PPV), and area under the curve (AUC) for the binary plaque burden cut-off. We additionally assessed the test characteristics for ACS of the plaque burden cut-off at various timepoints during follow-up [1, 2, 3, 5, 6.9 (median follow-up), and 10 years]. Kaplan–Meier curves were created and compared with the log-rank test. Additionally, subgroup analyses for age (below vs. above median age of 63 years) and sex were performed. The derived binary plaque burden cut-off was then tested in the external validation cohort. Analyses were two-tailed and a *P*-value of <0.05 was considered statistically significant. All analyses were performed with Stata version 15 (StataCorp. 2017. Stata Statistical Software: Release 15. College Station, TX: StataCorp LLC) and R version 4.3.2 (packages ‘survival’, ‘timeROC’, and ‘DTComPair’). S.B. and T.M. had full access to all the data in the study and take responsibility for its integrity and the data analysis.

## Results

### Patient characteristics and AI-QCT analysis in the derivation cohort

Hundred out of 2271 (4.4%) of patients experienced ACS (67 MI, 33 uAP) throughout a median follow-up of 6.9 (IQR 4.8–9.1) years. Patient characteristics and AI-QCT analysis of patients with vs. without ACS are reported in *Table 1*. Median time between CCTA and ACS was 1352 (IQR 502–2493) days. Overall, patients with vs. without ACS had higher plaque volumes and burden of all types (*P* < 0.001 for all) (*Table 1*).

**Table 1:** Patient baseline characteristics

Patient characteristics	Derivation cohort			Validation cohort		
	ACS (*n* = 100)	No ACS (*n* = 2171)	*P*-value	ACS (*n* = 27)	No ACS (*n* = 541)	*P*-value
Age, years	67 [60–72]	63 [56–69]	<0.001	58 [52–68]	59 [52–65]	0.258
Sex (male), *n* (%)	54 (54.0%)	901 (41.5%)	0.013	20 (74.1%)	294 (54.3%)	0.044
Hypertension, *n* (%)	75 (75.0%)	1213 (55.9%)	<0.001	16 (59.3%)	250 (46.2%)	0.185
Dyslipidaemia, *n* (%)	72 (72.0%)	1382 (63.7%)	0.089	12 (44.4%)	197 (36.4%)	0.398
Current smoker, *n* (%)	13 (13.0%)	274 (12.6%)	0.911	10 (37.0%)	180 (33.3%)	0.686
BMI, kg/m^2^	27.4 [24.9–30.0]	27.5 [24.7–31.2]	0.287	26.5 [24.8–30.1]	26.5 [24.3–29.1]	0.585
Diabetes mellitus, *n* (%)	17 (17.0%)	333 (15.3%)	0.653	6 (22.2%)	95 (17.6%)	0.536
Family history of CAD, *n* (%)	56 (56.0%)	1016 (46.8%)	0.072	14 (51.9%)	287 (53.1%)	0.903
Typical angina, *n* (%)	39 (39.0%)	488 (22.5%)	<0.001	13 (48.2%)	156 (28.8%)	0.032
Early elective revascularization (within 6 months) *n* (%)	27 (27.0%)	185 (8.5%)	<0.001	10 (37.0%)	104 (19.2%)	0.024
CACS, unit	329 [85–1257]	32 [0–241]	<0.001	321 [38–1024]	50 [0–303]	<0.001
Medication						
Antiplatelet drug (Aspirin or other), *n* (%)	59 (59.0%)	942 (43.4%)	0.002	24 (88.9%)	401 (74.1%)	0.084
Lipid-lowering drug, *n* (%)	45 (45.0%)	876 (40.4%)	0.354	18 (66.7%)	360 (66.5%)	0.989
Betablocker, *n* (%)	54 (54.0%)	962 (44.3%)	0.057	21 (77.8%)	326 (60.3%)	0.068
Long-acting nitrate, *n* (%)	14 (14.0%)	169 (7.8%)	0.026	12 (44.4%)	135 (25.0%)	0.024
Calcium channel blocker, *n* (%)	23 (23.0%)	321 (14.8%)	0.025	12 (44.4%)	135 (25.0%)	0.024
ACE inhibitor, *n* (%)	27 (27.0%)	373 (17.2%)	0.012	9 (33.3%)	98 (18.1%)	0.048
AT II antagonist, *n* (%)	25 (25.0%)	445 (20.5%)	0.277	5 (18.5%)	96 (17.7%)	0.918
AI-QCT						
AI-QCT diameter stenosis, %	56 [39–72]	21 [9–44]	<0.001	68 [53–77]	26 [9–60]	<0.001
CAD-RADS (based on AI-QCT diameter stenosis), n (%)			<0.001			<0.001
0	1 (1.0%)	149 (6.9%)		0 (0.0%)	23 (4.2%)	
1	13 (13.0%)	1043 (48.0%)		2 (7.4%)	240 (44.4%)	
2	18 (18.0%)	495 (22.8%)		4 (14.8%)	93 (17.2%)	
3	34 (34%)	265 (12.2%)		8 (29.6%)	92 (15.2%)	
4	29 (29.0%)	164 (7.6%)		11 (40.8%)	69 (12.7%)	
5	5 (5.0%)	55 (2.5%)		2 (7.4%)	34 (6.3%)	
Area stenosis, %	81 [62–92]	36 [15–70]	<0.001	89 [78–94]	44 [14–84]	<0.001
Remodelling index	1.5 [1.3–1.7]	1.3 [1.2–1.4]	<0.001	1.5 [1.4–1.6]	1.3 [1.2–1.5]	0.004
Vessel volume, mm^3^	3003 [2612–3664]	3048 [2509–3704]	0.999	2798 [2285–3374]	2577 [2097–3234]	0.218
Lumen volume, mm^3^	2538 [2068–3091]	2844 [2329–3440]	<0.001	2435 [1959–2776]	2317 [1915–2898]	0.994
Vessel length, mm	613 [534–681]	618 [546–687]	0.475	600 [543–653]	597 [525–651]	0.731
Total plaque volume, mm^3^	392 [149–806]	90 [28–269]	<0.001	422 [211–731]	121 [3–339]	<0.001
Non-calcified plaque volume, mm^3^	222 [123–385]	68 [25–173]	<0.001	252 [166–352]	84 [27–207]	<0.001
Low-attenuation plaque volume, mm^3^	0.0 [0.0–0.7]	0.0 [0.0–0.0]	<0.001	0.1 [0–2.1]	0.0 [0.0–0.4]	0.012
Calcified plaque volume, mm^3^	115 [27–364]	13 [0.1–86]	<0.001	81 [24–281]	19 [0–110]	<0.001
Percent atheroma volume, (%)	13.2 [6.0–23.2]	3.0 [1.0–7.1]	<0.001	13.6 [8.8–23.4]	4.6 [1.2–11.8]	<0.001
Percent non-calcified plaque volume, (%)	7.4 [4.4–11.6]	2.3 [0.9–5.5]	<0.001	8.3 [6.8–12.4]	3.3 [1.1–7.7]	<0.001
Percent low-attenuation plaque volume, (%)	0.0 [0.0–0.02]	0.0 [0.0–0.0]	<0.001	0.0 [0.0–0.6]	0.0 [0.0–0.1]	0.013
Percent calcified plaque volume, (%)	4.1 [1.0–10.4]	0.4 [0.0–2.7]	<0.001	3.8 [0.7–7.3]	0.7 [0.0–4.1]	<0.001
Two FPP, *n* (%)	48 (48.0%)	483 (22.3%)	<0.001	16 (59.3%)	187 (34.6%)	0.009

Values are *n* (%) or mean (±SD) or median [inter-quartile range]. *P*-values are from Mann–Whitney *U* test or χ^2^ test. Body mass index (BMI) was available for 82 patients with and 1374 without ACS in the derivation cohort, and for 26 with and 537 without ACS in the validation cohort. Coronary artery calcium score (CACS) was available for 83 patients with and 1781 patients without ACS in the derivation cohort, and for 27 patients with and 530 patients without ACS in the validation cohort.

AI-QCT, artificial intelligence-guided quantitative computed tomography; ACE, angiotensin converting enzyme; AP, angina pectoris; AT II, angiotensin II; CAD, coronary artery disease; CAD-RADS, Coronary Artery Disease-Reporting and Data System; two FPP, two feature positive plaque.

### Predictive value of plaque burden

The predictive value of continuous PAV, NCPV, and CPV per 1% increase on top of clinical characteristics (Reference 1), CAD-RADS (Reference 2), AI-QCT diameter stenosis ≥50%, (Reference 3), and two FPP (Reference 4) is shown in *Table 2*. For the model with two FPP, the added NCPV (%) did not include LD-NCPV (%), since this is already part of the two FPP definition. All plaque burden types remained independent predictors of ACS when added on top of all four reference models (*P* ≤ 0.006), except for NCPV (%) on top of two FPP (*P* = 0.093). According to the C-indexes, the addition of PAV or NCPV (%) significantly improved ACS prediction in all models (*P* ≤ 0.046), whereas CPV (%) did not further improve the prediction of ACS on top of CAD-RADS (*P* = 0.126) and AI-QCT diameter stenosis ≥50% (*P* = 0.324). The models including PAV showed the numerically highest C-indexes and performed in general similarly to models including only NCPV (%). Therefore, due to previously reported heterogeneity in NCPV (%) quantification across various software platforms (although predating AI-QCT),^19^ to improve generalizability of the study findings, PAV was chosen for cut-off derivation.

**Table 2:** Prognostic models for continuous plaque burden (derivation cohort)

2271 patients 100 ACS	Clinical (Reference 1)		PAV + Clinical		NCPV + Clinical		CPV + Clinical	
	HR (95% CI)	*P*-value	HR (95% CI)	*P*-value	HR (95% CI)	*P*-value	HR (95% CI)	*P*-value
Percent plaque volume, per 1%			1.05 (1.04–1.07)	<0.001	1.10 (1.06–1.14)	<0.001	1.06 (1.03–1.09)	<0.001
Age, per 1 year	1.05 (1.02–1.07)	<0.001	1.03 (1.01–1.06)	0.016	1.04 (1.01–1.06)	0.004	1.04 (1.01–1.06)	0.007
Sex (male vs. female)	1.55 (1.03–2.35)	0.037	1.22 (0.80–1.85)	0.363	1.11 (0.71–1.73)	0.644	1.44 (0.96–2.17)	0.080
Hypertension	2.03 (1.29–3.20)	0.002	1.67 (1.05–2.66)	0.029	1.73 (1.09–2.73)	0.020	1.80 (1.14–2.86)	0.012
Typical angina	1.82 (1.20–2.75)	0.005	1.70 (1.13–2.58)	0.012	1.73 (1.15–2.62)	0.009	1.75 (1.15–2.64)	0.008
Early revascularization^a^	2.27 (1.41–3.65)	0.001	1.28 (0.77–2.14)	0.344	1.27 (0.75–2.16)	0.373	1.63 (0.99–2.68)	0.055
C-index (95% CI)	0.744 (0.697–0.791)		0.798 (0.760–0.835)		0.799 (0.763–0.836)		0.770 (0.726–0.814)	
*P*-value for C-index difference between models	Reference 1		PAV vs. Ref 1	0.001	NCPV vs. Ref 1	0.001	CPV vs. Ref 1	0.032
			PAV vs. NCPV	0.819	NCPV vs. CPV	0.034	PAV vs. CPV	0.002

2271 patients, 100 ACS	CAD-RADS + Clinical (Reference 2)		PAV + CAD-RADS + Clinical		NCPV + +CAD-RADS + Clinical		CPV + CAD-RADS + Clinical	
	HR (95% CI)	*P*-value	HR (95% CI)	*P*-value	HR (95% CI)	*P*-value	HR (95% CI)	*P*-value
Percent plaque volume, per 1%			1.03 (1.01–1.05)	0.002	1.05 (1.01–1–10)	0.014	1.04 (1.01–1.06)	0.006
CAD-RADS	1.75 (1.47–2.09)	<0.001	1.53 (1.25–1.88)	<0.001	1.56 (1.27–1.92)	<0.001	1.64 (1.35–1.98)	<0.001
Age, per 1 year	1.03 (1.01–1.06)	0.012	1.03 (1.25–1.88)	0.051	1.03 (1.01–1.06)	0.019	1.03 (1.00–1.05)	0.053
Sex (male vs. female)	1.09 (0.71–1.67)	0.695	1.00 (0.65–1.54)	0.990	0.97 (0.62–1.51)	0.878	1.07 (0.69–1.64)	0.769
Hypertension	1.76 (1.12–2.78)	0.015	1.60 (1.00–2.53)	0.048	1.64 (1.03–2.59)	0.036	1.65 (1.04–2.61)	0.033
Typical angina	1.75 (1.16–2.64)	0.008	1.70 (1.13–2.57)	0.011	1.75 (1.16–2.63)	0.008	1.70 (1.12–2.57)	0.012
Early revascularization^a^	0.99 (0.58–1.67)	0.964	0.91 (0.54–1.54)	0.737	0.92 (0.55–1.55)	0.753	0.95 (0.56–1.60)	0.840
C-index (95% CI)	0.800 (0.7612–0.840)		0.811 (0.774–0.850)		0.809 (0.771–0.847)		0.807 (0.768–0.846)	
*P*-value for C-index difference	Reference 2		PAV vs. Ref 2	0.041	NCPV vs. Ref 2	0.025	CPV vs. Ref 2	0.126
			PAV vs. NCPV	0.600	NCPV vs. CPV	0.684	PAV vs. CPV	0.075

2271 patients 100 ACS	Clinical + AI-QCT stenosis ≥50% (Reference 3)		PAV + Clinical + AI-QCT stenosis ≥50%		NCPV + +Clinical + AI-QCT stenosis ≥50%		CPV + +Clinical + AI-QCT stenosis ≥50%	
	HR (95% CI)	*P*-value	HR (95% CI)	*P*-value	HR (95% CI)	*P*-value	HR (95% CI)	*P*-value
Percent plaque volume, per 1%			1.03 (1.01–1.05)	0.005	1.05 (1.01–1.10)	0.026	1.03 (1.00–1.06)	0.019
AI-QCT diameter stenosis ≥50%	4.85 (3.01–7.84)	<0.001	3.52 (2.05–6.05)	<0.001	3.70 (2.16–6.36)	<0.001	4.09 (2.46–6.79)	<0.001
Age, per 1 year	1.03 (1.00–1.06)	0.020	1.02 (0.99–1.05)	0.070	1.03 (1.00–1.05)	0.032	1.02 (0.99–1.05)	0.066
Sex (male vs. female)	1.14 (0.75–1.73)	0.549	1.04 (0.68–1.60)	0.847	1.01 (0.65–1.56)	0.964	1.12 (0.73–1.70)	0.607
Hypertension	1.76 (1.11–2.78)	0.016	1.61 (1.01–2.55)	0.045	1.65 (1.04–2.62)	0.033	1.66 (1.04–2.63)	0.033
Typical angina	1.77 (1.17–2.66)	0.007	1.72 (1.14–2.60)	0.009	1.75 (1.16–2.64)	0.008	1.73 (1.15–2.61)	0.009
Early revascularization^a^	1.10 (0.67–1.80)	0.705	0.99 (0.60–1.62)	0.956	0.98 (0.59–1.62)	0.940	1.05 (0.64–1.71)	0.851
C-Index (95% CI)	0.806 (0.767–0.844)		0.816 (0.779–0.852)		0.816 (0.780–0.852)		0.809 (0.771–0.847)	
*P*-value for C-index difference between models	Reference 3		PAV vs. Ref 3	0.046	NCPV vs. Ref 3	0.046	CPV vs. Ref 3	0.324
			PAV vs. NCPV	0.982	NCPV vs. CPV	0.269	PAV vs. CPV	0.052

2271 patients 100 ACS	Two FPP + Clinical (Reference 4)		PAV + two FPP + Clinical		NCPV^a^ + +two FPP + Clinical		CPV + two FPP + Clinical	
	HR (95% CI)	*P*-value	HR (95% CI)	*P*-value	HR (95% CI)	*P*-value	HR (95% CI)	*P*-value
Percent plaque volume, per 1%			1.05 (1.03–1.07)	<0.001	1.00 (1.00–1.00)	0.093	1.06 (1.03–1.08)	<0.001
Two FPP	1.97 (1.26–3.06)	0.003	1.38 (0.87–2.20)	0.173	1.81 (1.14–2.86)	0.012	1.74 (1.11–2.70)	0.015
Age, per 1 year	1.05 (1.02–1.07)	<0.001	1.03 (1.01–1.06)	0.014	1.03 (1.01–1.06)	0.025	1.03 (1.01–1.06)	0.007
Sex (male vs. female)	1.31 (0.85–2.02)	0.214	1.13 (0.74–1.75)	0.569	1.19 (0.76–1.86)	0.445	1.25 (0.82–1.92)	0.298
Hypertension	1.83 (1.15–2.90)	0.010	1.62 (1.02–2.58)	0.041	1.75 (1.10–2.79)	0.018	1.68 (1.05–2.67)	0.029
Typical angina	1.82 (1.20–2.75)	0.005	1.71 (1.13–2.60)	0.011	1.70 (1.11–2.60)	0.014	1.74 (1.15–2.63)	0.009
Early revascularization^a^	1.82 (1.11–2.98)	0.019	1.22 (0.73–2.04)	0.446	1.63 (0.97–2.75)	0.067	1.43 (0.87–2.37)	0.162
C-index (95% CI)	0.759 (0.716–0.803)		0.788 (0.749–0.826)		0.769 (0.725–0.812)		0.782 (0.742–0.823)	
*P*-value for C-index difference	Reference 4		PAV vs. Ref 4	<0.001	NCPV vs. Ref 4	0.003	CPV vs. Ref 4	0.038
			PAV vs. NCPV	0.013	NCPV vs. CPV	0.195	PAV vs. CPV	0.003

Multivariable Cox regressions for ACS. Clinical variables with a significant univariable association with ACS were included in the models (age, sex, hypertension, typical angina, and early revascularization within 6 months) (see Supplementary data online, *Table S1*).

^a^NCPV without LD-NCPV was added on top of two FPP, since LD-NCPV is part of the two FPP definition.

AI-QCT, artificial intelligence-guided quantitative coronary tomography; CAD-RADS, Coronary Artery Disease-Reporting and Data System; CI, confidence interval; HR, hazard ratio; CPV, calcified plaque volume; LD-NCPV, low-density non-calcified plaque volume; NCPV, non-calcified plaque volume; PAV, percent atheroma volume; two FPP, two feature positive plaque.

### Cut-off selection for PAV

**Figure 2:**
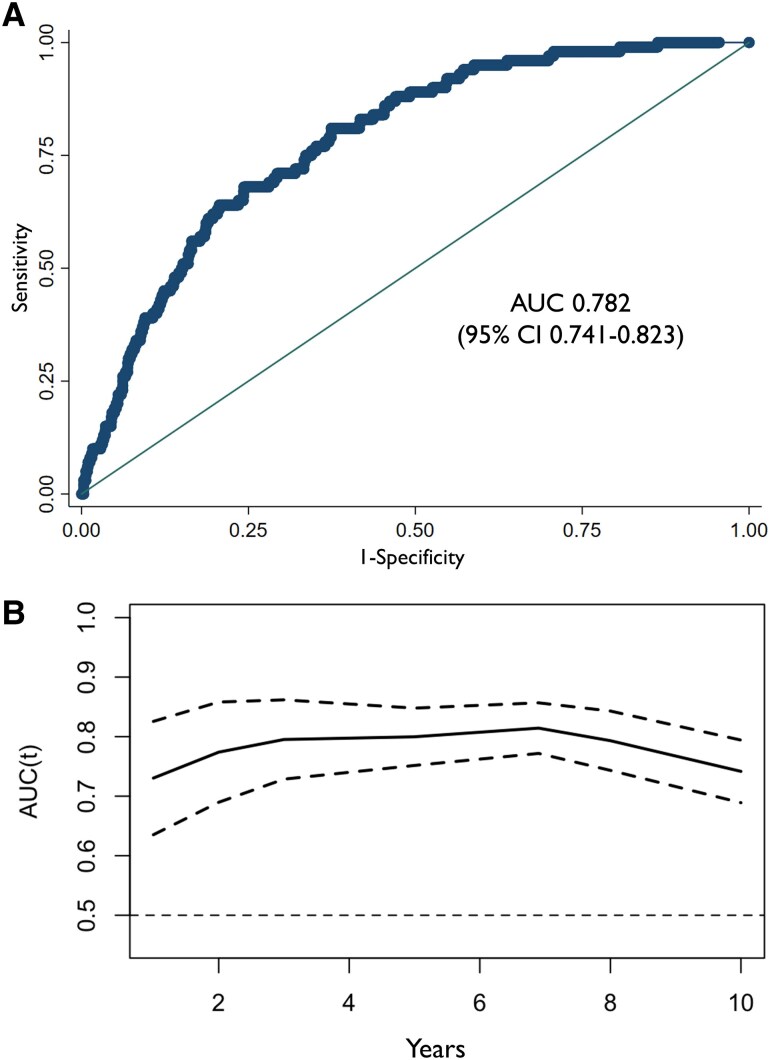
ROC analysis continuous PAV to predict ACS. (*A*) ROC analysis for continuous PAV to predict ACS in the derivation cohort (*n* = 2271). (*B*) Time-dependent AUC. Dashed lines represent 95% CI.

The AUC from ROC analysis of continuous PAV was 0.782 [95% confidence interval (CI) 0.743–0.823] (*Figure 2*). The cut-off with pre-defined ≥90% sensitivity for ACS was ≥2.6%. This cut-off resulted in a sensitivity of 90.0%, NPV of 99.0%, specificity of 46.8%, PPV of 7.2%, and AUC of 0.68 for ACS in the derivation cohort (*Graphical Abstract*, *Table 3*). Test characteristics of PAV 2.6% over time [at 1, 2, 3, 5, 6.9 (median follow-up), and 10] are shown in Supplementary data online, *Table S2*. A threshold selection according to the method of Youden in total plaque volume for comparison with previous work,^5^ resulted in 254.4 mm^3^. The test characteristics of this cut-off are shown in Supplementary data online, *Table S3*.

**Table 3:** Test characteristics of PAV 2.6%

	Derivation cohort	Validation cohort
Patients, *n*	2271	568
ACS, *n* (%)	100 (4.4%)	27 (4.8%)
Sensitivity, % (95% CI)	90.0 (82.4–95.1)	92.6 (75.7–99.1)
Specificity, % (95% CI)	46.8 (44.7–48.9)	35.7 (31.6–39.9)
PPV, % (95% CI)	7.2 (5.9–8.8)	6.7 (4.4–9.7)
NPV, % (95% CI)	99.0 (98.2–99.5)	99.0 (96.3–99.9)
AUC (95% CI)	0.68 (0.65–0.72)	0.64 (0.59–0.70)

ACS, acute coronary syndrome; AUC, area under the curve; CI, confidence interval; NPV, negative predictive value; PPV, positive predictive value.

### Predictive value of PAV 2.6% in the derivation cohort

**Figure 3:**
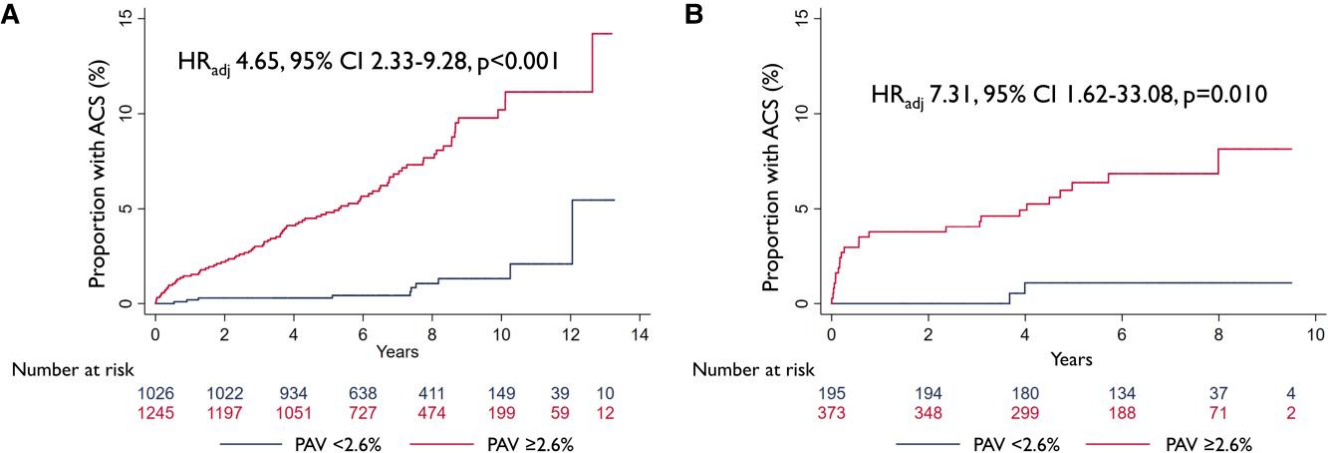
Kaplan–Meier curves for PAV 2.6%. Kaplan–Meier Curves for ACS in (*A*) the derivation and (*B*) the external validation cohort. HR_adj_, hazard ratio adjusted for age, sex, hypertension, and typical angina; PAV, percent atheroma volume.

Thousand hundred and forty-five out of 2271 patients (54.8%) had PAV ≥ 2.6% and 1026/2271 (45.2%) had PAV < 2.6%, which was markedly higher as compared with the 97/2271 (4.3%) patients with no plaque (PAV 0%) (*P* < 0.001). Ninety patients (7.2%) with PAV ≥ 2.6% as compared with 10 patients (1.0%) with PAV < 2.6% experienced ACS [Hazard ratio (HR) 7.68, 95% CI 4.00–14.77, *P* < 0.001] (*Graphical Abstract*, *Figure 3*, *Table 4*). This result was consistent after adjusting for clinical characteristics and early revascularization (HR 4.65, 95% CI 2.33–9.28, *P* < 0.001) (*Table 4*). Among the patients with PAV 0%, no ACS occurred. Subgroup analyses for age and sex showed consistent results (no interaction) with similar prognostic value of PAV 2.6% in women and men, however numerically higher prognostic value among patients of ≥63 years (median), even though confidence intervals (CI) were wide (see Supplementary data online, *Figure S1*).

**Table 4:** Univariable and multivariable cox regressions for PAV 2.6%

			Crude HR		Adjusted HR	
			HR (95% CI)	*P*-value	HR (95% CI)	*P*-value
Derivation 2271 patients 100 ACS	PAV ≥2.6% *n* = 1245	PAV < 2.6% *n* = 1026				
ACS, *n* (%)	90 (7.2%)	10 (1.0%)	7.68 (4.00–14.77)	<0.001	4.65 (2.33–9.28)^a^	<0.001^a^
Validation 568 Patients 27 ACS	PAV ≥ 2.6% *n* = 373	PAV < 2.6% *n* = 195				
			
ACS, *n* (%)	25 (6.7%)	2 (1.0%)	7.94 (1.87–33.62)	0.005	7.31 (1.62–33.08)^b^	0.010^b^

Results were adjusted for variables with significant univariable associations with ACS (see Supplementary data online, *Table S1*). For the validation cohort, due to the limited number of events, the variables with the strongest associations were included.

^a^Age, sex, hypertension, typical angina pectoris, and early revascularization.

^b^Typical angina and early revascularization.

ACS, acute coronary syndrome; CI, confidence interval; HR, hazard ratio; PAV, percent atheroma volume.

### Patient characteristics in the external validation cohort

In the external validation cohort, 27/568 (4.8%) of patients experienced ACS (19 MI, 8 uAP) throughout a median follow-up of 6.7 (IQR 4.7–7.8) years. Median time between CCTA and ACS was 282 (IQR 51–1473) days. Patient characteristics and AI-QCT analysis of patients with vs. without ACS are shown in *Table 1*.

### External validation of PAV ≥ 2.6%

In the external validation cohort, 373/568 patients (65.7%) had PAV ≥ 2.6% and 195/568 (34.3%) had PAV < 2.6%, which was significantly higher as compared with the 15/568 (2.6%) patients with no plaque (PAV 0%) (*P* < 0.001). The diagnostic test characteristics of PAV ≥ 2.6% to detect ACS were sensitivity 92.6%, NPV 99.0%, specificity 35.7%, PPV 6.7%, and AUC 0.64 (*Graphical Abstract*, *Table 3*).

Twenty-five patients (6.7%) with PAV ≥ 2.6% as compared with 2 patients (1.0%) with PAV <2.6% experienced ACS (HR 7.94, 95% CI 1.87–33.62, *P* = 0.005) (*Graphical Abstract*, *Figure 3*, *Table 4*). This result remained consistent after adjusting for typical angina and early revascularization (HR 7.31, 95% CI 1.62–33.08, *P* = 0.010) (*Table 4*). Other variables were not included due to the limited numbers of events. No ACS occurred among patients with PAV 0%.

### Sensitivity analyses

A sensitivity analysis excluding patients who underwent early elective revascularization showed consistent results (see Supplementary data online, *Tables S3* and *S4*). Test characteristics and prognostic value of PAV 2.6% for MI alone as well as the composite of ACS or all-cause death are reported in Supplementary data online, *Tables S5* and *S6*.

## Discussion

In this observational cohort study on patients who underwent CCTA for suspected CAD, we assessed the association between AI-based plaque burden quantification and ACS, and aimed to derive and validate a plaque burden safety threshold for long-term ACS. We found that AI-QCT plaque burden showed a consistent and continuous association with ACS throughout a median follow-up of 7 years on top of clinical predictors, CAD-RADS, obstructive stenosis, and two FPP. A threshold of PAV 2.6% was derived to identify future ACS patients with 90.0% sensitivity in the derivation cohort. This cut-off showed a sensitivity of 92.6% in the validation cohort and excellent NPV of 99.0% in both the derivation and validation cohort. Importantly, 43% of the patients could be identified as having low long-term risk of ACS based on PAV < 2.6% in both cohorts, as compared with only 4% of patients showing absence of plaque (PAV 0%) based on AI-QCT. PAV ≥ 2.6% showed strong prognostic value with five- to seven-fold increased adjusted rates of ACS in both cohorts.

### Plaque burden and the risk of ACS

Numerous prior studies have demonstrated the association between plaque burden on CCTA and ACS.^20–22^ However, lesion-based studies have generally shown poor PPV for ACS, since a majority of vulnerable plaques will rupture silently without causing a direct ischaemic event for a given patient.^23^ Consequently, the concept of the vulnerable patient has evolved.^24^ Therefore, in this study, we aimed for a patient-level plaque burden cut-off that takes into account the totality of coronary atherosclerosis on CCTA. In line with the previous findings using major cardiovascular events as endpoint,^7^ we found independent prognostic value of plaque burden quantified by AI-QCT on top of clinical characteristics, CAD-RADS, obstructive stenosis, and two FPP. Among the different plaque types, PAV showed the numerically highest C-indexes and performed similarly to NCPV (%), whereas CPV (%) did not consistently improve event prediction. Therefore, and since NCPV (%) quantification was reported to vary across software platforms (although predating AI-QCT),^19^ to improve generalizability of the study findings, PAV was chosen for cut-off derivation.

Conventional clinical CCTA analysis is based on semi-quantitative measures of plaque burden and manual QCT has been available for many years, but remained restricted to research situations, because it is time-consuming. Technological developments during recent years with AI can finally make plaque quantification feasible and widely available for clinical use. Complete quantification and characterization of the whole coronary tree are now possible within ∼10 min, and together with final human quality control and report generation a total time of ∼25–30 min per scan are needed.^4^ Together with its potential to improve risk stratification,^7,8^ as also evidenced in this study, and diagnostic accuracy comparable to the consensus of three Level III readers,^4^ AI-guided CCTA analysis represents a promising next step in CCTA technology. However, whereas conventional CCTA represents the current state-of-the art imaging modality to rule-out significant CAD,^1,2^ AI-guided analysis detects any plaque (PAV > 0%) in up to 96–97% of patients.^7^ The clinical implications of these observations were unknown so far. Therefore, we aimed to derive and validate a clinically useful safety cut-off for AI-based PAV regarding long-term coronary events. However, our current study does not aim to prove superiority of AI- vs. non-AI-based CCTA analysis.

### A binary plaque burden cut-off for ACS risk stratification

In a substudy of the SCOT-HEART trial (Scottish COmputed Tomography of the HEART Trial), a cut-off for low-attenuation plaque burden of 4% was identified, which was associated with 4.7-fold elevated adjusted risk of future MI throughout 5 years.^21^ More recently, using a deep-learning approach for coronary plaque quantification, a cut-off for total plaque volume of 238.5 mm^3^ was derived and shown to be associated with a 7.3-fold higher crude risk of MI.^5^ However, for both cut-off derivations, the method of Youden was applied, which is not suitable for ruling out adverse patient outcome, where more weight to sensitivity must be given.

In the current study, we aimed to determine a patient-level safety cut-off for AI-based plaque burden, to predict future ACS with a pre-defined sensitivity of ≥90%. This resulted in a cut-off of PAV 2.6%. Since we used this sensitivity-focussed approach, it was expected that the cut-off would be lower as previously suggested.^21^ For conceptual validation with previous work,^5^ we also tested a threshold according to Youden in total plaque volume resulting in a cut-off of 254.4 mm^3^, close to the Youden-based cut-off of 238.5 mm^3^ determined to detect MI with another AI-based plaque quantification tool.^5^ For our cut-off selection, to account for different plaque volumes across different patient sizes, also related to patients’ sex, we had chosen plaque burden normalized to the total vessel volume, instead of absolute plaque volume. Subgroup analyses yielded indeed similar progntaostic value among women vs. men, however markedly higher prognostic value for patients >63 years, potentially related to their higher event rate in general, although CI were wide and the result may be interpreted with caution.

### Safety cut-off for ACS

The main aim of this cut-off derivation was to identify patients at low risk for ACS. Patients below the cut-off had indeed good prognosis with only 1% cumulative incidence of ACS over a median follow-up of 7 years. Forty-five per cent of patients in the derivation and 34% of patients in the validation cohort were classified at low risk according to this cut-off (PAV < 2.6%), whereas the vast majority of patients (96%) had any plaque detected by AI-QCT (PAV > 0%). Our analyses may thus suggest that a plaque burden up to the derived and validated threshold of PAV 2.6% is associated with low rates of ACS, and may therefore not justify intensified downstream testing or allocation to advanced atherosclerotic medication. This information may be helpful for the reasonable clinical application of AI-guided plaque analysis, which detects any plaque in up to 96–97% of patients. Furthermore, in relation to a previously validated plaque staging system based on PAV,^7^ it may allow for further subdivision of stage 1 (PAV > 0–5%) into patients with low event risk with PAV < 2.6% vs. those with relatively increased risk with PAV ≥ 2.6%.

### Limitations

The results of this study must be considered in the light of several limitations. It represents an observational study with all the inherent limitations of a non-randomized investigation. However, this was in part mitigated by the use of two independent patient cohorts with large to moderate sample size and long follow-up. Data collection was performed in part retrospectively, however CCTA scans were re-analysed by AI-QCT in 2022–23 blinded to clinical data and outcomes, and events adjudicated appropriately. The CCTA scans were performed with relatively old CT scanners and newer generation scanners might provide better image quality. However, analysis of such older scans allowed for long-term follow-up, a key issue in the current analysis. For this analysis, the prescription rate based upon the CCTA findings of antiplatelet agents, statins, and other medication with potential outcome benefit were not available. However, for the derivation cohort from Turku, statin initiation and its association with clinical outcomes has been reported previously.^25^ Data on risk factor management after CCTA (e.g. lipid levels) were not available. With the focus on mechanistic links between predictors and outcome, our study endpoint included only acute coronary events and no other events related to atherosclerosis. This ACS endpoint was limited to non-fatal events, since data on cardiac causes of death was not available. However, focussing on less severe (i.e. non-fatal) events could potentially have rendered an even lower (safer) threshold to classify patients at low risk for ACS. Furthermore, we have addressed this by performing a sensitivity analysis on the composite of ACS or all-cause death. The derivation and validation cohort differed with respect to the occurrence of ACS after CCTA with later occurrence in the Turku and earlier occurrence in the Amsterdam cohort. However, given the consistent main results of this study, this may even reinforce the robustness of the derived cut-off and its ability to safely rule-out ACS over mid- and long-term in varying cohorts. Whereas 99.9% of patients completed follow-up in the derivation cohort, 9% of patients were lost to follow-up in the external validation cohort. Patients underwent coronary revascularization as clinically indicated, which may have affected the outcome. However, we have accounted for this by adjusting the models for early revascularization and performing a sensitivity analysis excluding patients who underwent early revascularization, showing consistent findings with the main analysis.

## Conclusions

This study suggests that a PAV up to 2.6% quantified by AI is associated with low-ACS risk in two independent patient cohorts. This PAV cut-off may be helpful for clinical application of AI-guided CCTA analysis, by classifying 43% of patients at low risk for ACS, whereas AI detects any plaque in up to 96–97% of patients.

## Supplementary Material

jeaf121_Supplementary_Data

## Data Availability

Data will be made available upon reasonable request from the corresponding author.
