# Supplementary material for: Derivation and validation of an artificial intelligence-based plaque burden safety cut-off for long-term acute coronary syndrome from coronary computed tomography angiography
Source: Eur Heart J Cardiovasc Imaging. 2025 Apr 17;26(7):1163–73. doi: 10.1093/ehjci/jeaf121 (PMC12206583; doi:10.1093/ehjci/jeaf121)
Supplement: jeaf121_Supplementary_Data [file jeaf121_supplementary_data.docx]

**SUPPLEMENTAL MATERIAL**

**Table S1. Univariable Cox Regressions for ACS**

|  | **DERIVATION COHORT**  **2271 Patients, 100 ACS** | | **VALIDATION COHORT**  **568 Patients, 27 ACS** | |
| --- | --- | --- | --- | --- |
|  | **HR (95% CI)** | **p-value** | **HR (95% CI)** | **p-value** |
| **Age, per 1 year** | 1.05 (1.03-1.08) | <0.001 | 1.02 (0.98-1.06) | 0.293 |
| **Sex (male vs. female)** | 1.56 (1.05-2.31) | 0.026 | 2.51 (1.06-5.94) | 0.036 |
| **Hypertension** | 2.37 (1.51-3.73) | <0.001 | 1.68 (0.78-3.61) | 0.188 |
| **Diabetes mellitus** | 1.18 (0.70-2.00) | 0.525 | 1.13 (0.44-2.91) | 0.801 |
| **Dyslipidemia** | 1.40 (0.91-2.17) | 0.128 | 1.36 (0.64-2.91) | 0.428 |
| **Smoking** | 1.47 (0.98-2.18) | 0.059 | 1.13 (0.52-2.48) | 0.752 |
| **Family history of CAD** | 1.44 (0.97-2.14) | 0.069 | 0.98 (0.46-2.08) | 0.950 |
| **Typical angina** | 1.73 (1.15-2.61) | 0.009 | 2.35 (1.10-4.99) | 0.027 |
| **Early revascularization** | 3.34 (2.14-5.22) | <0.001 | 2.83 (1.28-6.24) | 0.010 |
| **CAD-RADS** | 1.90 (1.65-2.18) | <0.001 | 1.89 (1.44-2.49) | <0.001 |
| **AI-QCT ≥50% stenosis** | 6.67 (4.38-10.15) | <0.001 | 8.44 (2.18-22.41) | <0.001 |
| **Two FPP** | 3.05 (2.06-4.52) | <0.001 | 3.17 (1.43-6.98) | 0.004 |
| **PAV, per 1%** | 1.07 (1.06-1.09) | <0.001 | 1.04 (1.02-1.07) | <0.001 |
| **NCPV, per 1%** | 1.13 (1.10-1.17) | <0.001 | 1.05 (1.02-1.08) | <0.001 |
| **CPV, per 1%** | 1.09 (1.07-1.11) | <0.001 | 1.06 (1.02-1.11) | 0.007 |

ACS = acute coronary syndrome, AI-QCT = artificial intelligence-guided quantitative computed tomography, CAD = coronary artery disease, CAD-RADS = Coronary Artery Disease-Reporting and Data System, CI = confidence interval, CPV = calcified plaque volume, HR = hazard ratio, NCPV = non-calcified plaque volume, PAV = percent atheroma volume, two FPP = two feature positive plaque.

**Table S2. Test Characteristics of PAV ≥2.6% for ACS Over Time (Derivation Cohort)**

|  | **1 Year** | **2 Years** | **3 Years** | **5 Years** | **6.9 Years (median FUP)** | **10 Years** |
| --- | --- | --- | --- | --- | --- | --- |
| **ACS, N** | 20 | 30 | 40 | 60 | 76 | 95 |
| **Sensitivity, %**  **(95% CI)** | 90.0  (76.8-100) | 90.0  (79.3-100) | 92.5  (84.3-100) | 95.2  (89.9-100) | 95.1  (90.3-99.9) | 90.5  (93.8-97.2) |
| **Specificity, %**  **(95% CI)** | 45.8  (43.7-47.9) | 46.1  (44.0-48.2) | 46.6  (44.5-4.7) | 47.1  (44.7-49.5) | 47.3  (44.4-50.2) | 42.8  (37.6-48.0) |
| **PPV, %**  **(95% CI)** | 1.5  (0.8-2.2) | 2.2  (1.4-3.0) | 3.0  (2.0-4.0) | 4.8  (3.6-6.0) | 6.8  (5.2-8.4) | 9.5  (7.2-11.8) |
| **NPV, %**  **(95% CI)** | 99.8  (99.5-100) | 99.7  (99.4-100) | 99.7  (99.4-100) | 99.7  (99.4-100) | 99.6  (99.2-100) | 98.6  (97.5-99.7) |
| **AUC**  **(95% CI)** | 0.68  (0.61-0.75) | 0.68  (0.63-0.74) | 0.70  (0.65-0.74) | 0.71  (0.68-0.74) | 0.71  (0.68-0.74) | 0.67  (0.62-0.71) |

ACS = acute coronary syndrome, AUC = area under the curve, CI = confidence interval, FUP = follow-up, NPV = negative predictive value, PAV = percent atheroma volume, PPV = positive predictive value.

**Table S3. Additional Test Characteristics for ACS**

|  | **DERIVATION COHORT** | | **VALIDATION COHORT** | |
| --- | --- | --- | --- | --- |
|  | **Total plaque volume**  **≥254.4mm^3^**  **(Youden threshold)** | **PAV**  **≥2.6%**  **(Sensitivity Analysis*)** | **Total plaque volume**  **≥254.4mm^3^**  **(Youden threshold)** | **PAV**  **≥2.6%**  **(Sensitivity Analysis*)** |
| **Patients, N** | 2271 | 2059 | 568 | 454 |
| **Sensitivity, %**  **(95% CI)** | 68.0  (57.9-77.0) | 86.3  (76.2-93.2) | 66.7  (46.0-83.5) | 88.2  (63.6-98.5) |
| **Specificity, %**  **(95% CI)** | 73.7  (71.7-75.5) | 50.8  (48.6-53.0) | 68.9  (64.9-72.8) | 43.2  (38.5-48.0) |
| **PPV, %**  **(95% CI)** | 10.6  (8.3-13.3) | 6.1  (4.7-7.7) | 9.7  (5.8-14.9) | 5.7  (3.2-9.2) |
| **NPV, %**  **(95% CI)** | 98.0  (97.2-98.7) | 99.0  (98.2-99.5) | 97.6  (95.6-98.9) | 99.0  (96.3-99.9) |
| **AUC**  **(95% CI)** | 0.71  (0.66-0.76) | 0.69  (0.64-0.73) | 0.68  (0.59-0.77) | 0.66  (0.58-0.74) |

*The sensitivity analysis excluded 212 patients in the derivation cohort and 114 patients in the validation cohort who underwent early elective revascularization within 6 months. ACS = acute coronary syndrome, AUC = area under the curve, CI = confidence interval, NPV = negative predictive value, PAV = percent atheroma volume, PPV = positive predictive value.

**Table S4. Sensitivity Analysis Excluding Patients with Early Elective Revascularization**

| **DERIVATION**  **2059 Patients**  **73 ACS** | **PAV ≥2.6%**  **N=1040** | **PAV**  **<2.6%**  **N=1019** | **Crude HR** | | **Adjusted HR** | |
| --- | --- | --- | --- | --- | --- | --- |
|  |  |  | **HR (95% CI)** | **p-value** | **HR (95% CI)** | **p-value** |
| **ACS, n (%)** | 63 (6.1%) | 10 (1.0%) | 6.48 (3.33-12.64) | <0.001 | 4.08 (2.03-8.21)^1^ | <0.001^1^ |
| **VALIDATION**  **454 Patients**  **17 ACS** | **PAV**  **≥2.6%**  **N=263** | **PAV**  **<2.6%**  **N=191** | **Crude HR** | | **Adjusted HR** | |
|  |  |  | **HR (95% CI)** | **p-value** | **HR (95% CI)** | **p-value** |
| **ACS, n (%)** | 15 (5.7%) | 2 (1.1%) | 6.72 (1.49-30.28) | 0.013 | 7.14 (1.54-33.07)^2^ | 0.012^2^ |

The sensitivity analysis excluded 212 patients in the derivation cohort and 114 patients in the validation cohort who underwent early elective revascularization within 6 months. Results were adjusted for variables with significant univariable associations (**Table S1**). For the validation cohort, due to the limited number of events, only the strongest predictors were included. 1) age, sex, hypertension, typical angina pectoris; 2) typical angina pectoris. ACS = acute coronary syndrome, CI = confidence interval, HR = hazard ratio, PAV = percent atheroma volume.

**Table S5. Test Characteristics PAV ≥2.6% for Other Clinical Endpoints**

|  | **DERIVATION COHORT** | | **VALIDATION COHORT** | |
| --- | --- | --- | --- | --- |
| **Patients, N** | 2271 | | 568 | |
| **Event, N** | **MI**  **N=68** | **ACS or Death**  **N=255** | **MI**  **N=19** | **ACS or Death**  **N=50** |
| **Sensitivity, %**  **(95% CI)** | 88.2  (78.1-94.8) | 83.9  (78.8-88.2) | 89.5  (66.9-98.7) | 96.0  (86.3-99.5) |
| **Specificity, %**  **(95% CI)** | 46.2  (44.1-48.3) | 48.9  (46.7-51.1) | 35.2  (31.2-39.3) | 37.3  (33.1-41.6) |
| **PPV, %**  **(95% CI)** | 4.8  (3.7-6.2) | 17.2  (15.1-19.4) | 4.6  (2.7-7.2) | 12.9  (9.6-16.7) |
| **NPV, %**  **(95% CI)** | 99.2  (98.5-99.7) | 96.0  (94.6-97.1) | 99.0  (96.3-99.9) | 99.0  (96.3-99.9) |
| **AUC**  **(95% CI)** | 0.67  (0.63-0.71) | 0.66  (0.64-0.69) | 0.62  (0.55-0.70) | 0.67  (0.63-0.70) |

ACS = acute coronary syndrome, AUC = area under the curve, CI = confidence interval, MI = myocardial infarction, NPV = negative predictive value, PAV = percent atheroma volume, PPV = positive predictive value.

**Table S6. Sensitivity Analysis Other Clinical Endpoints**

| **DERIVATION** | **PAV ≥2.6%**  **N=1245** | **PAV**  **<2.6%**  **N=1026** | **Crude HR** | | **Adjusted HR** | |
| --- | --- | --- | --- | --- | --- | --- |
|  |  |  | **HR (95% CI)** | **p-value** | **HR (95% CI)** | **p-value** |
| **MI, n (%)** | 60 (4.8%) | 8 (0.9%) | 6.32 (3.02-13.21) | <0.001 | 3.98 (1.83-8.67)^1^ | 0.001^1^ |
| **Death or ACS, n (%)** | 214 (17.2%) | 41 (4.0%) | 4.46 (3.20-6.24) | <0.001 | 2.78 (1.94-3.98)^2^ | <0.001^2^ |
| **VALIDATION** | **PAV**  **≥2.6%**  **N=373** | **PAV**  **<2.6%**  **N=195** | **Crude HR** | | **Adjusted HR** | |
|  |  |  | **HR (95% CI)** | **p-value** | **HR (95% CI)** | **p-value** |
| **MI, n (%)** | 17 (4.6%) | 2 (1.0%) | 4.88 (1.13-21.14) | 0.034 | 4.62 (1.06-20.05)^3^ | 0.041^3^ |
| **Death or ACS, n (%)** | 48 (12.9%) | 2 (1.0%) | 15.43 (3.71-64.16) | <0.001 | 8.94 (2.08-38.39)^4^ | 0.003^4^ |

Results were adjusted for variables with significant univariable associations with the respective endpoint: 1) age, hypertension, smoking, dyslipidemia, typical angina pectoris, early revascularization within 6 months. 2) age, sex, hypertension, smoking, diabetes mellitus, typical angina, early revascularization within 6 months. 3) typical angina. 4) age, sex, diabetes mellitus, early revascularization within 6 months. ACS = acute coronary syndrome, CI = confidence interval, HR = hazard ratio, MI = myocardial infarction, PAV = percent atheroma volume.

**Figure S1. Subgroup Analyses for Age and Sex (Derivation Cohort)**

**
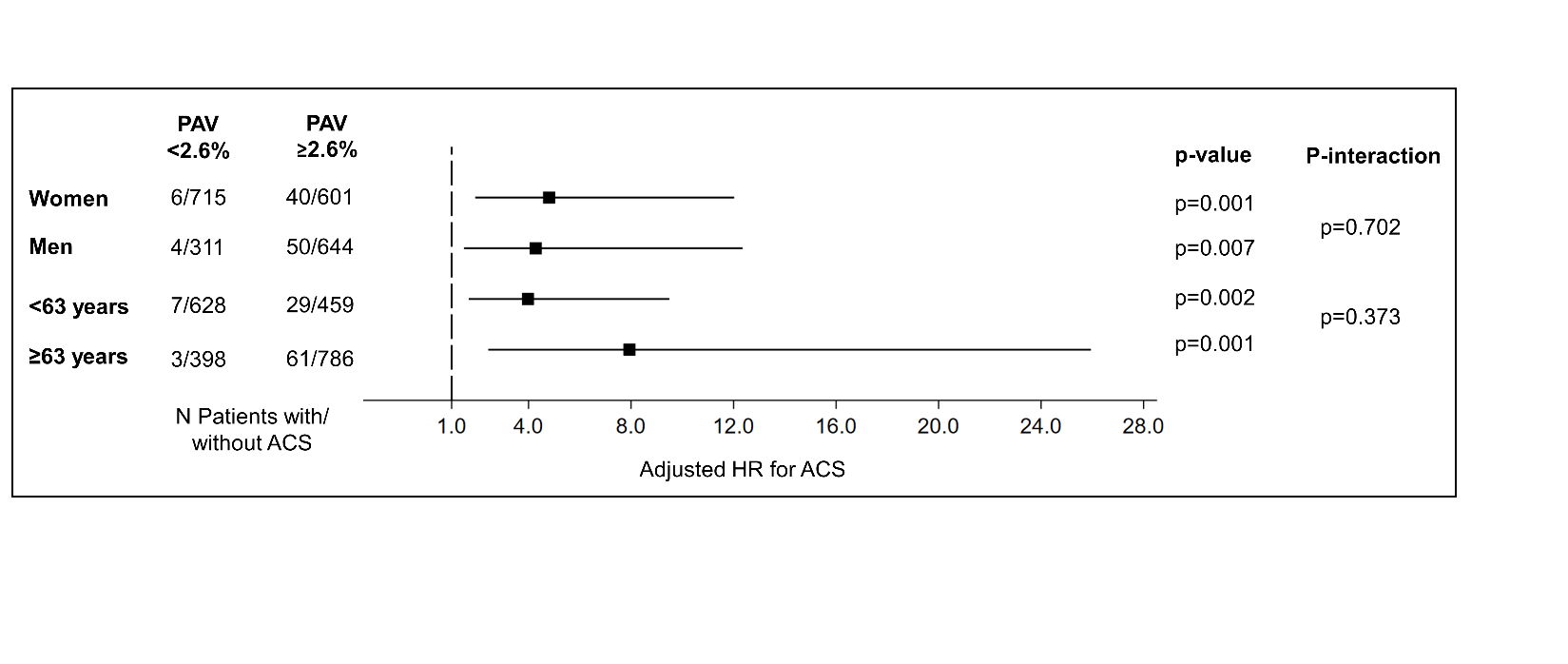
**

Hazard ratios (HR) were adjusted for age (only subgroup sex), sex (only subgroup age), hypertension, typical angina, and early revascularization within 6 months. ACS = acute coronary syndrome, PAV = percent atheroma volume.
